# Supplementary material for: Exogenous Application of dsRNA for Protection against Tomato Leaf Curl New Delhi Virus
Source: Viruses. 2024 Mar 12;16(3):436. doi: 10.3390/v16030436 (PMC10974794; doi:10.3390/v16030436)
Supplement: Supplementary file 1 [file viruses-16-00436-s001.zip › SupplementaryFigureS1_Frascati_et_al_Viruses_Revised.pptx]

## Slide 1
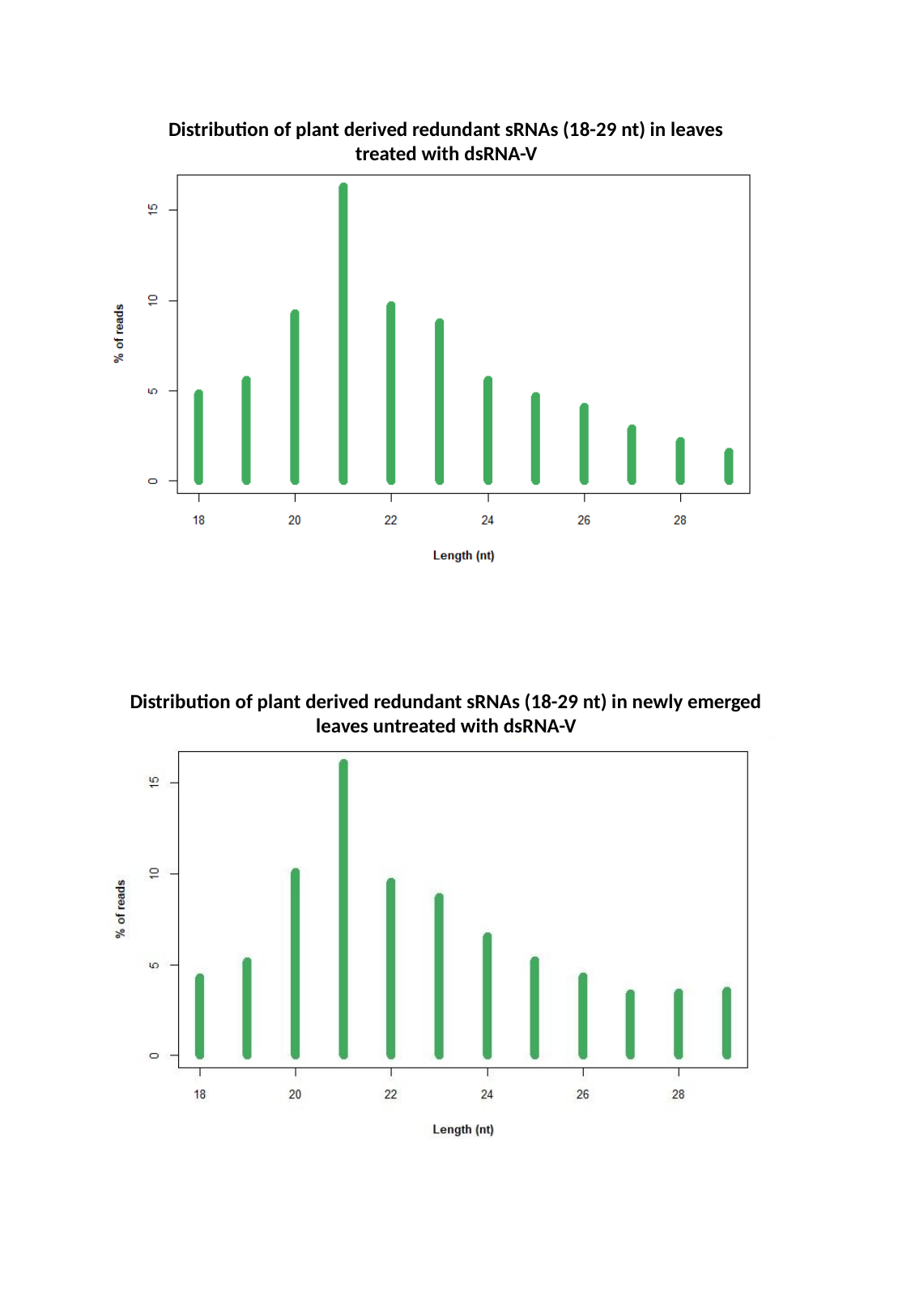

Distribution of plant derived redundant sRNAs (18-29 nt) in leaves treated with dsRNA-V
Distribution of plant derived redundant sRNAs (18-29 nt) in newly emerged leaves untreated with dsRNA-V

## Slide 2
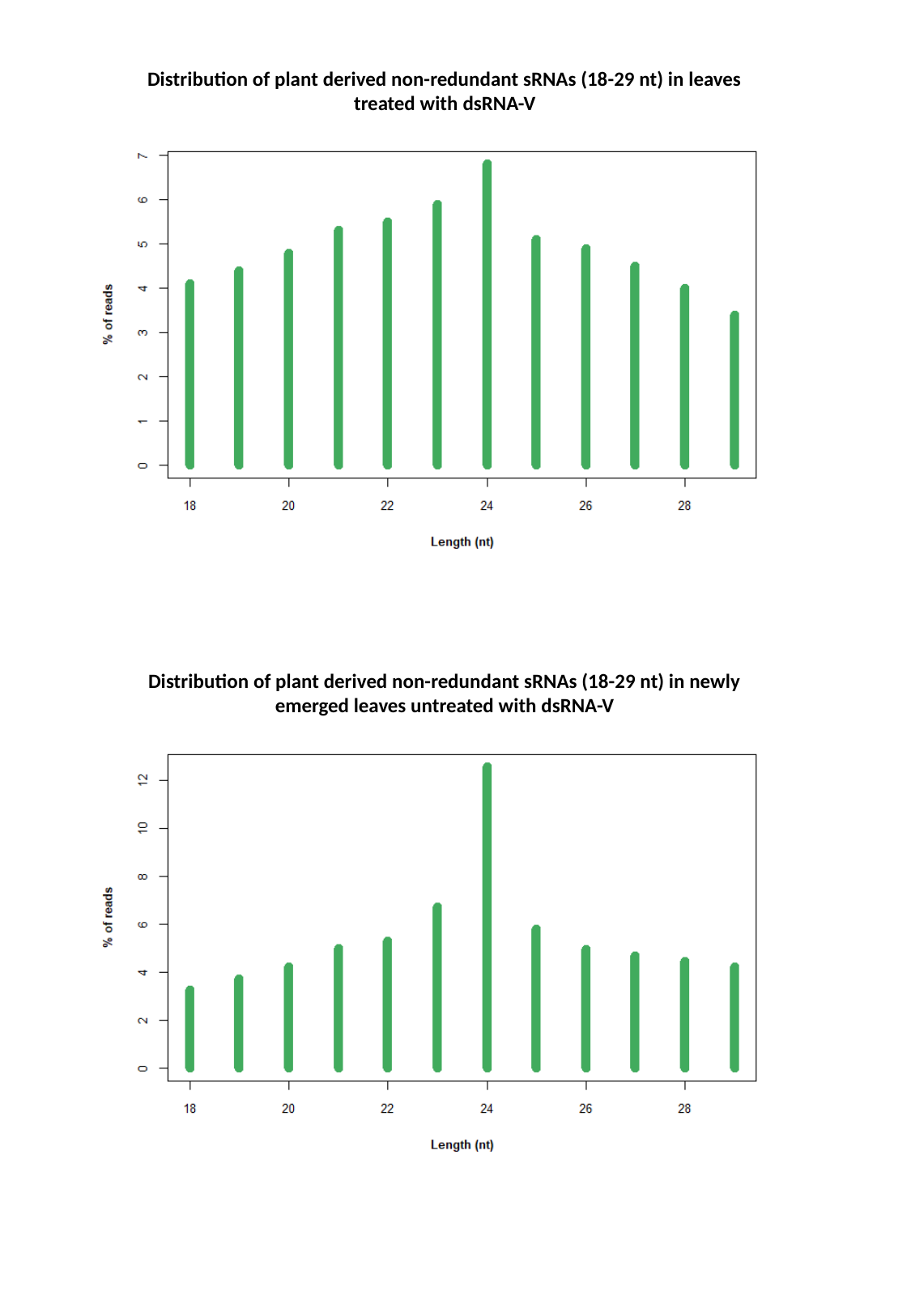

Distribution of plant derived non-redundant sRNAs (18-29 nt) in leaves treated with dsRNA-V
Distribution of plant derived non-redundant sRNAs (18-29 nt) in newly emerged leaves untreated with dsRNA-V
