# Supplementary material for: Exogenous Application of dsRNA for Protection against Tomato Leaf Curl New Delhi Virus
Source: Viruses. 2024 Mar 12;16(3):436. doi: 10.3390/v16030436 (PMC10974794; doi:10.3390/v16030436)
Supplement: Supplementary file 1 [file viruses-16-00436-s001.zip › SupplementaryTableS2_Frascati_et_al_Viruses_Revised.pdf]

Supplementary Table 2. Reads statistics

	raw reads	clean reads	reads mapping on dsRNA-V	% reads mapping on dsRNA-V
**treated leaves**	87,457,181	72,844,452	2,589,756	3.56
**newly emerged untreated leaves**	59,665,201	46,276,817	318	0.00069
