# Supplementary material for: Exogenous Application of dsRNA for Protection against Tomato Leaf Curl New Delhi Virus
Source: Viruses. 2024 Mar 12;16(3):436. doi: 10.3390/v16030436 (PMC10974794; doi:10.3390/v16030436)
Supplement: Supplementary file 1 [file viruses-16-00436-s001.zip › SupplemetaryTableS1_Frascati_et_al_Viruses_revised.pdf]

Supplementary Table 1. List of primers used in this work.

Name	Sequence (5' to 3')	Reference
dsND_endPCR_fw	ATGTATGCCTTGACGTCCGA	This work
dsND_endPCR_rv	GATTCACGCACAGGGGAGTA	This work
dsND_qPCR_fw	CGTAAGCCCGTCCAGATTTG	This work
dsND_qPCR_rv	TGCCTGACCAATCACGACAT	This work
qToLCNDV_B_fw	TCCAAGGATTCTTATCCTTKAGAGAG	Luigi et al., 2020
qToLCNDV_B_rv	CAAGCAGAATTCACAATTCCAATC	Luigi et al., 2020
ZuEF-1A_fw	GCTTGGGTGCTCGACAAACT	Obrero et al., 2011
ZuEF-1A_rv	TCCACAGAGCAATGTCAATGG	Obrero et al., 2011
